# Use of sanger and next-generation sequencing to screen for mosaic and intronic *APC* variants in unexplained colorectal polyposis patients

**DOI:** 10.1007/s10689-021-00236-2

**Published:** 2021-03-08

**Authors:** Fadwa A. Elsayed, Carli M. J. Tops, Maartje Nielsen, Hans Morreau, Frederik J. Hes, Tom van Wezel

**Affiliations:** 1 Department of Pathology, Leiden University Medical Center, Leiden, the Netherlands; 2 Department of Clinical Genetics, Leiden University Medical Center, Leiden, the Netherlands

**Keywords:** Unexplained colorectal polyposis, *APC*, Mosaic variants, Intronic variants, Pseudoexons

## Abstract

In addition to classic germline *APC* gene variants, *APC* mosaicism and deep intronic germline *APC* variants have also been reported to be causes of adenomatous polyposis. In this study, we investigated 80 unexplained colorectal polyposis patients without germline pathogenic variants in known polyposis predisposing genes to detect mosaic and deep intronic *APC* variants. All patients developed more than 50 colorectal polyps, with adenomas being predominantly observed. To detect *APC* mosaicism, we performed next-generation sequencing (NGS) in leukocyte DNA. Furthermore, using Sanger sequencing, the cohort was screened for the following previously reported deep intronic pathogenic germline *APC* variants: c.1408 + 731C > T, p.(Gly471Serfs*55), c.1408 + 735A > T, p.(Gly471Serfs*55), c.1408 + 729A > G, p.(Gly471Serfs*55) and c.532-941G > A, p.(Phe178Argfs*22). We did not detect mosaic or intronic *APC* variants in the screened unexplained colorectal polyposis patients. The results of this study indicate that the deep intronic *APC* variants investigated in this study are not a cause of colorectal polyposis in this Dutch population. In addition, NGS did not detect any further mosaic variants in our cohort.

## Introduction

Pathogenic germline variants in *APC* (MIM# 611,731) cause familial adenomatous polyposis syndrome (FAP; MIM# 175,100), a rare autosomal dominant-inherited syndrome characterized by the development of multiple colorectal adenomas and a very high risk of colorectal cancer [1–4]. In classic FAP, patients develop hundreds to thousands of colorectal adenomatous polyps, while in attenuated FAP (AFAP), patients develop fewer adenomas (< 100) at a later age than those with classical FAP [5–8]. A subset of patients with multiple colorectal adenomas and no *APC* germline variants have been found to carry biallelic variants in the base excision repair gene *MUTYH* (MIM# 604,933), causing *MUTYH*-associated polyposis (MAP; MIM# 608,456) [9]. In addition, a number of other genes associated with adenomatous polyposis, such as *POLE*, *POLD1*, *NTHL1*, *MSH3* and *MLH3*, have recently been reported [10–13]. The detection rate of *APC* variants in FAP patients depends on phenotype and methods. In classic FAP, *APC* germline variants can be detected in up to 85% of patients [14, 15]; however, the detection rates of *APC* germline variants in patients with fewer colorectal adenomatous polyps (AFAP patients) are lower, ranging from 10 to 30% of patients [14, 16], suggesting that a proportion of pathogenic variants remain undetected by routine methods [17–19]. Mosaic *APC* variants and deep intronic variants localized in regions not covered by PCR-based diagnostics were previously identified as additional causal factors. Using RNA-based assays and next-generation sequencing (NGS), it has been shown that a proportion of variant-negative FAP patients harbor molecular changes in deep intronic regions of *APC* [19, 20]. These studies identified deep intronic *APC* variants that result in pseudoexon formation [19, 20]. Through the use of sensitive techniques, somatic *APC* mosaicism has been demonstrated in a minority of adenomatous polyposis patients [21–26]. In addition, using deep sequence analysis of *APC* in DNA isolated from multiple adenomas, mosaic variants were identified in 9 of 18 patients with 21 to 100 adenomas; in some of these cases, NGS also detected the variants in leukocyte DNA at low frequency [27]. In this study, we investigate the role of deep intronic germline *APC* variants and mosaic *APC* variants in leukocyte DNA as possible genetic causes of colorectal polyposis in a Dutch cohort of unexplained patients with more than 50 polyps.

## Materials and methods

### Patients

A total of 80 index patients with more than 50 colorectal polyps (Table 1) were selected from a previously described cohort [28–31]. The cohort included patients previously screened for germline mosaic *APC* variants by denaturing gradient gel electrophoresis (DGGE) [17], the protein truncation test (PTT) [17] and high resolution melting analysis (HRMA) [21]. All cases tested negative for pathogenic germline variants in *APC*, *MUTYH*, *POLE*, and *POLD1* and for *NTHL1* hotspot variants. Clinicopathological data included date of birth, gender, age at diagnosis of colorectal polyps/adenomas, cumulative number of polyps, location and histology of polyps/adenomas, information on CRC and presence of polyps/CRC in first-degree family members. Since the term serrated adenomas is currently preferred over hyperplastic polyps, we lumped together polyps described as such under the term sessile serrated lesions with or without dysplasia. Three controls were included in this study. Leukocyte DNA from this cohort was available for the study. The study was approved by the medical ethics committee of Leiden University Medical Center, protocol P01-019.

**Table 1:** Clinical characteristics of the colorectal polyposis patients (n = 80)

Patient characteristics	Individuals %
Number of polyps	
> 100	29 (36.2%)
50–100	51 (63.8%)
Type of polyps	
Adenomas	36 (45%)
Mixed (Adenomas + Serrated*)	38 (47.5%)
Serrated	5 (6.2%)
Unknown	1 (1.3%)
Age at diagnosis with polyposis	
≥ 50 years	49 (61.3%)
< 50 years	31 (38.7%)
Diagnosed with CRC	
Yes	27 (33.8%)
No	53 (66.2%)
Age at diagnosis with CRC	
> 50	19 (70.4%)
≤ 48	8 (29.6%)
Sex	
Male	53 (66.2%)
Female	27 (33.8%)
Polyposis family	
Polyposis family	29
No polyposis family	37
Unknown	14
CRC family	
CRC family	33
No CRC family	34
Unknown	13

^*^Sessile serrated lesions with or without dysplasia

### *APC* intronic variant screening

Leukocyte DNA of the patients was screened for the intronic *APC* variants in Table 2 using Sanger sequencing. Primers were designed using Primer3 software http://primer3.ut.ee/ and were obtained from Eurofins Genomics (Ebersberg, Germany). The following primers with universal M13 tails were used: c.1408 + 731C > T, c.1408 + 735A > T and c.1408 + 729A > G; forward: 5′-TGTAAAACGACGGCCAGTATCATGCTGAACCATCTCAT-3′ and reverse: 5′ CAGGAAACAGCTATGACCAAATGACGAATGAAACGATG-3′. For c.532-941G > A; forward: 5′ TGTAAAACGACGGCCAGTAGAGGGTTTGGGAAGTGGAG-3′ and reverse: 5′ CAGGAAACAGCTATGACCTCTGTGTGCCCTTAGAAAACTG-3′. Sanger sequencing of the PCR amplified fragments was performed by Macrogen (Amsterdam, Netherlands). The sequencing results were analyzed using Mutation Surveyor software (Sofgenetics, State College PA, USA).

**Table 2:** Summary of the germline pathogenic *APC* intronic variants

Intron	Alteration in genomic DNA	Insertion length (bp)	RNA alteration	Predicted protein alteration	Publication
4	c.532-941G > A	Insertion of 167 bp	r.531_532ins532-1106_532-940	p.Phe178Argfs*22	[19]
10	c.1408 + 731C > T	Insertion of 83 bp	r.1408_1409ins1408 + 647_1408 + 729	p.Gly471Serfs*55	[19, 20]
10	c.1408 + 735A > T	Insertion of 83 bp	r.1408_1409ins1408 + 647_1408 + 729	p.Gly471Serfs*55	[19]
10	c.1408 + 729A > G	Insertion of 83 bp	r.1408_1409ins1408 + 647_1408 + 729	p.Gly471Serfs*55	[20]

### Next-generation sequencing and data analysis

Deep *APC* sequencing was performed using a previously described custom *APC* panel [27]. The complete sequencing panel consisted of 115 amplicons (11,216 bp), covering 99.3% of the coding regions of *APC*. Libraries were prepared with Ion Ampliseq™ 2.0 Kit (Thermo Fisher Scientific, Bleiswijk, The Netherlands) according to the manufacturer’s instructions and were sequenced on the Ion Torrent Proton Platform (Thermo Fisher Scientific, Bleiswijk, The Netherlands). Sequence data were analyzed as described previously [27]. Variants were annotated to the GenBank reference sequence NM_000038.4. The Integrative Genomics Viewer (IGV) (https://www.broadinstitute.org/igv/) was used to visualize the read alignment and the presence of variants against the reference genome.

## Results and discussion

In this study, we attempt to identify the genetic causes of colorectal polyposis in unexplained patients with colorectal polyposis. Deep NGS of *APC* was performed to identify possible undetected pathogenic mosaic variants. Furthermore, *APC* intronic germline variants described previously [19, 20] were studied to evaluate their role. A high-risk cohort was selected for this study, consisting of 80 index patients with ≥ 50 colorectal polyps (Table 1), of whom many had a relatively early onset, which increases the probability of finding undiscovered mosaic or intronic variants. The mean age at diagnosis of colorectal polyps was 49 years (range 12–80). The majority of patients (n = 51, 63.8% with a mean age of 51 years at diagnosis) had a cumulative polyp count between 50 and 100, while 29 patients (36.2% with a mean age of 46 years at diagnosis) showed more than 100 polyps. Forty-five percent of the patients displayed only adenomatous polyps, while 47.5% of the patients displayed a mixed phenotype with adenomas and sessile serrated lesions with or without dysplasia. CRC was found in 27 patients (33.8%, with a mean age of 56 years, range 37–80). The clinical characteristics of the patients are summarized in Table 1.

First, we screened the leukocyte DNA of 80 patients for the following deep intronic heterozygous germline variants in *APC:* c.1408 + 731C > T, p.(Gly471Serfs*55), c.1408 + 735A > T, p.(Gly471Serfs*55), c.1408 + 729A > G, p.(Gly471Serfs*55) and c.532-941G > A, p.(Phe178Argfs*22). We did not detect any of these variants in our cohort. The study by Spier et al. [19] was the first to describe *APC*-related pseudoexons in FAP patients from Germany. These pseudoexons were caused by three heterozygous germline variants with a combined frequency of 6.4% (8/125); *APC* c.532-941G > A was identified in five patients, *APC* c.1408 + 731C > T was identified in two patients, and *APC* c.1408 + 735A > T was identified in one patient [19]. In a second study by Nieminen et al. [20], two additional intronic variants were identified in a cohort of 54 patients from Finland: *APC* c.1408 + 729A > G and *APC* c.646-1806 T > G and the variant identified previously by Spier et al., *APC* c.1408 + 731C > T. The overall reported frequency of these variants from the study by Nieminen et al. was 5.5% (3/54). The reported frequency of these intronic variants from both studies is approximately 6%. Nevertheless, we could not detect these variants in our cohort, possibly because either the frequency of intronic variants is lower in the Dutch population and the sample size of our cohort is not large enough or because these variants are local founder variants.

**Fig. 1:**
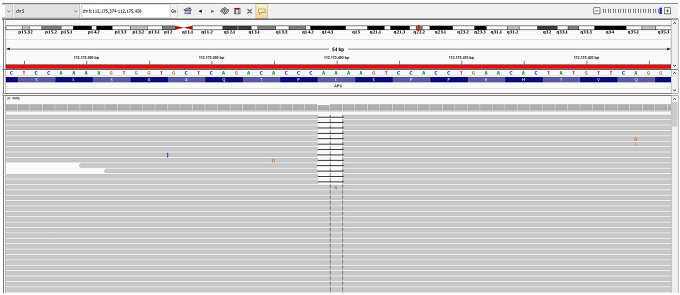
Integrative Genomics Viewer (IGV) images of next-generation sequencing (NGS) data of mosaic *APC* c.4110_4111delAA variant detected in the leukocyte DNA of the positive control sample

Subsequently, we performed deep *APC* sequencing of leukocyte DNA from 80 colorectal polyposis patients. Our positive controls were two previously described cases with mosaic *APC* variants [27]; *APC* c.4110_4111delAA was reported to be present in leukocyte DNA with 4% variant allele frequency (VAF), and *APC* c.2493dupA was reported with a VAF of 3% in leukocyte DNA. The *APC* mosaic variant c.4057G > T served as a negative control, as the variant was detected previously [27] in normal colonic mucosa and was absent in leukocyte DNA. Both positive controls, *APC* c.4110_4111delAA (Fig. 1) and *APC* c.2493dupA, were clearly present, while *APC* c.4057G > T was absent in the negative control. No additional *APC* mosaic variants were detected in our cohort. A limitation of this study is that we used only leukocyte DNA for mosaicism screening due to the scarcity of available DNA from patient adenomas. Mosaicism might remain undetectable or be overlooked if the molecular analysis is limited to blood, even when sensitive techniques are applied, due to very low or even absent presentation of the mutated allele [23, 27]. Peripheral blood cells arise from the mesoderm, and when the variant occurs after mesoderm and endoderm specification (early postzygotic mutation), the mosaicism is likely restricted to the colon and is difficult to detect the variant in leukocyte DNA [23, 27, 32, 33]. In a previous study, it was recommended to test at least two or more adenomas to detect mosaic variants [27].

A recent systematic review of current *APC* mosaicism studies recommends testing adenomas from the polyposis patients without *APC* germline variant to allow the detection of low allele frequency mosaicism as well as mosaicism confined to colon [33]. Consequently, in our study, *APC* mosaic variants confined to the colon could have been missed because we could not screen the DNA from the adenomas of the patients.

In conclusion, we did not detect any of the four previously reported *APC* intronic variants in our cohort. We also did not detect mosaic *APC* variants in our cohort using deep sequencing analysis in blood. This finding suggests that the benefit of using targeted amplicon-based NGS to further scrutinize the *APC* gene in unexplained cases of polyposis is limited. Analyzing DNA from adenomas in addition to leukocyte DNA is recommended to detect a possible underlying mosaicism. Also, other approaches, such as whole genome sequencing or transcriptome sequencing, could be employed to detect undiscovered intronic or promoter variants or other regulatory variants.

## Abbreviations

FAP: Familial adenomatous polyposis syndrome
AFAP: Attenuated FAP
NGS: Next-generation sequencing
DGGE: Denaturing gradient gel electrophoresis
PTT: Protein truncation test
HRMA: High resolution melting analysis
IGV: Integrative genomics viewer
VAF: Variant allele frequency
